# Lipase From *Rhizomucor miehei* Immobilized on Magnetic Nanoparticles: Performance in Fatty Acid Ethyl Ester (FAEE) Optimized Production by the Taguchi Method

**DOI:** 10.3389/fbioe.2020.00693

**Published:** 2020-06-30

**Authors:** Katerine da S. Moreira, André L. B. de Oliveira, Lourembergue S. de M. Júnior, Rodolpho R. C. Monteiro, Thays N. da Rocha, Fernando L. Menezes, Lillian M. U. D. Fechine, Juliano C. Denardin, Sebastian Michea, Rafael M. Freire, Pierre B. A. Fechine, Maria C. M. Souza, José C. S. dos Santos

**Affiliations:** ^1^Departamento de Engenharia Química, Universidade Federal do Ceará, Campus do Pici, Fortaleza, Brazil; ^2^Instituto de Engenharias e Desenvolvimento Sustentável, Universidade da Integração Internacional da Lusofonia Afro-Brasileira, Campus das Auroras, Redenção, Brazil; ^3^Group of Chemistry of Advanced Materials (GQMat) – Department of Analytical Chemistry and Physic-chemistry, Federal University of Ceará – UFC, Fortaleza, Brazil; ^4^Departamento de Física/Center for the Development of Nanoscience and Nanotechnology (CEDENNA), Universidad de Santiago de Chile (USACH), Santiago, Chile; ^5^Institute of Applied Chemical Sciences, Universidad Autónoma de Chile, Santiago, Chile

**Keywords:** lipase from *Rhizomucor miehei*, immobilized, magnetic nanoparticles, APTES, fatty acid ethyl ester, Taguchi

## Abstract

In this communication, it was evaluated the production of fatty acid ethyl ester (FAAE) from the free fatty acids of babassu oil catalyzed by lipase from *Rhizomucor miehei* (RML) immobilized on magnetic nanoparticles (MNP) coated with 3-aminopropyltriethoxysilane (APTES), Fe_3_O_4_@APTES-RML or RML-MNP for short. MNPs were prepared by co-precipitation coated with 3-aminopropyltriethoxysilane and used as a support to immobilize RML (immobilization yield: 94.7 ± 1.0%; biocatalyst activity: 341.3 ± 1.2 U*_p_*_–NPB_/g), which were also activated with glutaraldehyde and then used to immobilize RML (immobilization yield: 91.9 ± 0.2%; biocatalyst activity: 199.6 ± 3.5 U*_p_*_–NPB_/g). RML-MNP was characterized by X-Ray Powder Diffraction (XRPD), Fourier Transform-Infrared (FTIR) spectroscopy and Scanning Electron Microscope (SEM), proving the incorporation and immobilization of RML on the APTES matrix. In addition, the immobilized biocatalyst presented at 60°C a half-life 16–19 times greater than that of the soluble lipase in the pH range 5–10. RML and RML-MNP showed higher activity at pH 7; the immobilized enzyme was more active than the free enzyme in the pH range (5–10) analyzed. For the production of fatty acid ethyl ester, under optimal conditions [40°C, 6 h, 1:1 (FFAs/alcohol)] determined by the Taguchi method, it was possible to obtain conversion of 81.7 ± 0.7% using 5% of RML-MNP.

## Introduction

Lipases represent the most widely used class of enzymes in biotechnological applications and organic chemistry (Javed et al., 2018; Pinheiro et al., 2018; Souza et al., 2020), due to some unique properties, such as selectivity and mild reaction conditions (Miranda et al., 2014). In fact, lipases are used in different areas, such as biofuel production (Rodrigues and Fernandez-Lafuente, 2010; de Vasconcellos et al., 2018; Sahoo et al., 2018). Among the renewable fuels, biodiesel stands out as one of the most promising (Wenlei and Ning, 2009; Christopher et al., 2014; Okoro et al., 2019).

Biodiesel is a renewable fuel made from biomass, such as plants (vegetable oils) or animals (animal fat) (dos Santos Silva et al., 2011; Chua et al., 2020; Singh et al., 2020). It is a mixture of methyl or ethyl esters of fatty acids (Tiwari et al., 2018; Zhong et al., 2020), produced by the transesterification and esterification reaction in the presence of a catalyst (Teo et al., 2016; Aguieiras et al., 2017), such as lipases (Ycel et al., 2012; Verdasco-Martín et al., 2016; Okoro et al., 2019; Moreira et al., 2020).

Despite the high catalytic efficiency of lipases, factors linked to stability and cost limit the use of these biocatalysts (Fernandez-Lopez et al., 2017). In this sense, the immobilization of lipases is used as a tool for enzyme for favoring recovery and reuse (Brady and Jordaan, 2009; Rodrigues et al., 2015; dos Santos et al., 2017) besides, it promotes an improvement in enzyme activity, selectivity or specificity, stability and purity, aside resistance to such inhibitors (Barbosa et al., 2013, 2015; Rios et al., 2018; Monteiro et al., 2019a; Bezerra et al., 2020).

One strategy for lipase immobilization is the interfacial activation on hydrophobic supports (Adlercreutz, 2013; Lima et al., 2015; Manoel et al., 2015; Reis et al., 2019; Rodrigues et al., 2019). This method allows, among other things, the immobilization, modulation, and stabilization of the enzyme in a single step (Cunha et al., 2014; Manoel et al., 2016). However, at high temperatures or in organic media, lipase molecules may be released from the support (Fernandez-Lorente et al., 2011; Hirata et al., 2016a, b); besides, the desorption of the lipase from the support may also be caused by the surfactant properties of some substrates and reaction products (Virgen-Ortíz et al., 2017).

Magnetic nanoparticles have highlighted among many nanoparticles of distinct materials due to the great possibility of modifying its magnetic properties with the effects of size and large surface area (Xie and Ma, 2009; Karimi, 2016). When used as supports to immobilization, magnetic nanoparticles present as advantages the possible recovery of the enzymatic derivative, by magnetic separation, allowing reuse in several production (dos Santos et al., 2015a; Karimi, 2016; Rodrigues et al., 2019). Through the functionalization of the surface, intended to facilitate the occurrence of the enzyme-support bond, the introduction of chemical groups necessary for the immobilization of enzymes is carried out (Neto et al., 2017; Lee et al., 2019).

In this sense, lipase from *Rhizomucor miehei* (RML) has some applications in the hydrolysis of oils in free fatty acid and glycerol (Rodrigues and Fernandez-Lafuente, 2010). For instance, it was reported that RML selectively hydrolyzed saturated fatty acid in soybean oil, but had no effect in relation to epoxidized soybean oil (Wang and Schuman, 2013). Furthermore, esterification reactions were catalyzed by RML through solid-state fermentation with growth in babassu cake, with a conversion of over 80% with the use of palm and soy fatty acids (Aguieiras et al., 2017). Therefore, RML has been widely used due to its properties, such as specificity in the release of fatty acids, catalytic activity, and stability (Babaki et al., 2015; dos Santos et al., 2015b).

In order to optimize the production of fatty acid ethyl ester from the free fatty acids of babassu oil catalyzed by RML immobilized on magnetic nanoparticles, the Taguchi method was used. Developed by Taguchi and Konishi, the Taguchi method (also known as an orthogonal matrix design) is a useful tool to be applied in industrial processes (Adewale et al., 2017). Remarkably, for biochemical processes, it is possible to optimize processes and increase the quality of products (Lesovik et al., 2018; Fediuk et al., 2019; Murali and Fediuk, 2020), with a reduced number of experimental runs and, therefore, reduced costs (Adewale et al., 2017). Improving quality and reliability is made through design, and this requires proper planning and layout of the experiments and accurate analysis of the results (Murali and Fediuk, 2020).

## Materials and Methods

### Materials

The commercial extract of lipase from *Rhizomucor miehei* (RML) (5.9 mg of protein/mL) was a kind gift from Novozymes (Spain). 3-aminopropyltriethoxysilane (APTES), glutaraldehyde solution grade II 25% (w/v), and *p*-nitrophenyl butyrate (*p-*NPB) and Triton X-100, were purchased from Sigma-Aldrich (Sigma-Aldrich, St Louis, MI, United States); ethanol (P.A. 99.96%) from Dinâmica (São Paulo, Brazil). Iron magnetic nanoparticles (Fe_3_O_4_) were produced by the co-precipitation method. The chemical reagents used for this synthesis were FeCl_3_.6H_2_O (pure granulated 99%), FeSO_4_.7H_2_O (pure granulated 99%) and 30% ammonia solution, supplied by Sigma-Aldrich (Sigma-Aldrich, Saint Louis, MI, United States).

### Methods

#### Synthesis of Iron Magnetic Nanoparticles (Fe_3_O_4_) Functionalized With 3-Aminopropyltriethoxysilane (APTES)

The magnetic nanoparticles functionalized with APTES were produced following the methodology described by Monteiro et al. (2019a). In 30 mL of deionized water, 2.5 g of FeSO_4_^.^7H_2_O (9 mmol) and 4.0 g of FeCl_3_^.^6H_2_O (15 mmol) were dissolved, under mechanical stirring for 30 min at 60°C. Forty milliliters of concentrated ammonium hydroxide were added to the iron cation solution. The system remained under mechanical agitation at a temperature of 60°C for 30 min. After that time, the nanoparticles were washed several times with deionized water and three times with ethanol. Subsequently, the non-functionalized Fe_3_O_4_ nanoparticles were poured into 300 mL of ethanol (95%) and placed in an ultrasonic bath (37 kHz and 300 W) for 1 h at room temperature. Then 10 mL of APTES was added to the mixture. The system remained for another hour in the ultrasonic bath and after that time, the functionalized nanoparticles were washed four times with ethanol and dried under vacuum (Monteiro et al., 2019b).

#### Activation of Fe_3_O_4_@APTES With Glutaraldehyde (GLU)

The nanoparticles of Fe_3_O_4_@APTES were activated with glutaraldehyde (Fe_3_O_4_@APTES-GLU), to provide the covalent bond between the enzyme and the support. For this, 0.1 g of Fe_3_O_4_@APTES previously dried was put in contact with 100 μL of glutaraldehyde. The mixture remained under constant stirring for 1 h at 25°C. Then, the mixture was washed three times with sodium phosphate buffer solution (25 mM and pH 7) to remove excess glutaraldehyde (Xie and Ma, 2009; Monteiro et al., 2019b).

#### Covalent Immobilization of RML Onto Fe_3_O_4_@APTES-GLU

RML was immobilized on Fe_3_O_4_@APTES-GLU, called Fe_3_O_4_@APTES-GLU-RML, by covalent bonding. For this, 0.1 g of Fe_3_O_4_@APTES-GLU were suspended in 1 mL of sodium phosphate buffer solution (25 mM and pH 7) containing RML (enzymatic load: 10 mg/g support) in the presence of 0.01% Triton X-100. The system was placed under constant agitation for 1 h at 25°C. After that time, the biocatalyst was separated from the solution by magnetic decanting and washed with sodium phosphate buffer solution (25 mM and pH 7) until neutrality. To determine the amount of enzyme immobilized on the support, the initial and final concentration of RML in the supernatant of the immobilization suspension was measured (Bezerra et al., 2017; Monteiro et al., 2019a). The protein concentration was determined using the method described by Bradford (1976) and bovine serum albumin was used as a reference (Bradford, 1976). For the reduction of Schiff’s bases reduction, after the enzyme immobilization step, 1.0 mg.mL^–1^ sodium borohydride was added to the immobilization suspension and kept under agitation during 30 min at 25°C. After this, the derivative was filtered and thoroughly rinsed with 0.2 M buffer phosphate pH 7.0 and finally washed thoroughly with distilled water (Mendes et al., 2011).

#### Adsorption Immobilization of RML on Fe_3_O_4_@APTES

RML was immobilized on Fe_3_O_4_@APTES, called Fe_3_O_4_@APTES-RML (RML-MNPA), by adsorption. For this, 0.1 g of Fe_3_O_4_@APTES were suspended in 1 mL of sodium phosphate buffer solution (25 mM and pH 7) containing RML (enzymatic load: 10 mg/g support) (Monteiro et al., 2019a). The system was placed under constant agitation for 1 h at 25°C. After that time, the biocatalyst was separated from the solution by magnetic decanting and washed with sodium phosphate buffer solution (25 mM and pH 7) until neutrality. To determine the amount of enzyme immobilized on the support, the initial and final concentration of RML in the supernatant of the immobilization suspension was measured (Bezerra et al., 2017; Monteiro et al., 2019b). The protein concentration was determined using the method described by Bradford (1976) and bovine serum albumin was used as a reference (Bradford, 1976).

#### Determination of Enzymatic Activity and Protein Concentration

The hydrolytic activity of soluble and immobilized RML was performed following the methodology described by Rios et al. (2016). Lipase activity was determined by increasing the absorbance at 348 nm produced by the hydrolysis of *p*-NPB as a substrate during 90 s, under magnetic stirring. The analyzes were performed in a 25 mM sodium phosphate buffer solution at pH 7 and 25°C (ε in these conditions is 5150 M^–1^ cm^–1^) (de Souza et al., 2016). To initiate the reaction, 50 μL of suspended lipase solution was added to 50 μL of *p*-NPB and 2.5 mL of the buffer solution, to initiate the reaction. Under these conditions, an international unit of activity (U) was defined as the amount of enzyme that hydrolyzes 1 μmol of *p*-NPB per minute. The protein concentration was determined using the method described by Bradford (1976) and bovine serum albumin was used as a reference (Bradford, 1976).

#### Immobilization Parameters

Immobilization parameters were evaluated according to the methodology described by Pinheiro et al. (2019). Immobilization yield (IY) was defined as the percentage of enzymatic activity that was immobilized, that is, the ratio between the activity of the enzymes retained in the support (initial activity – final activity) and initial activity. The theoretical activity (At_T_) of lipase immobilized on the support can be calculated using the amount of enzyme offered per g support and the immobilization yield (dos Santos et al., 2017). And the recovered or expressed activity (At_R_) was defined as the ratio between biocatalyst activity (At_D_) and theoretical activity (At_T_).

#### Characterization of the Obtained Materials

X-ray powder diffraction (XRPD) patterns of the synthesized nanoparticles were collected using a Bruker D2 Phaser diffractometer, controlled by a Diffract. measurement software, operating at 30 kV and 10 mA in Bragg-Brentano reflection geometry with CoKα radiation (λ = 1.7880 Å), using a 2° range 20°–90° and a scanning rate of 2° min^–1^. The functionalization, chemical modification of the support and immobilization process of all samples were carried out by FTIR spectroscopy. The spectra were obtained for dried samples (pressed in disk-shaped KBr pellet) in the range 4000–400 cm^–1^ using a Perkin Elmer 2000 spectrophotometer. Transmission electron microscopy (TEM) images of biocatalyst were obtained using a Hitachi^®^ HT7700 TEM system operating at an accelerating voltage of 120 kV. In order to perform the TEM investigation, the nanomaterials were firstly dispersed in ethanol and deposited onto a carbon-coated copper grid sample holder. The magnetic curves were obtained using a vibrating sample magnetometer (VSM) at 300 K. In order to assure the magnetic moments values acquired, the VSM was previously calibrated using a standard reference material (yttrium Iron Garnet Sphere) from the National Institute of Standards and Technology (NIST). For all measurements, the magnetic moment obtained for each applied field was normalized by the mass of NPs.

#### Effect of pH on Biocatalyst Activity

To analyze the effect of pH on the activity of the biocatalyst, free and immobilized lipase were resuspended in 1 mL of 25 mM buffer in the pH range ranging from 5 to 10 [sodium acetate (pH range 3.6–5.6), sodium phosphate (pH range 5.8–8.0), and sodium carbonate (pH range 8.9–10.8)] and using *p*-NPB as described earlier. The enzyme was incubated in each buffer for 15 min, and then the activity was measured (Monteiro et al., 2019a).

#### Thermal Stability

The thermal stability of free and immobilized lipase was determined by incubation in sodium acetate buffer (25 mM; pH 5), sodium phosphate buffer (25 mM; pH 7), or sodium carbonate buffer (25 mM; pH 10), at a temperature of 60°C. The activity of the samples was measured periodically using *p*-NPB and sodium phosphate buffer (25 mM; pH 7), the residual activity was expressed as a percentage of the initial activity (hydrolytic activity before thermal incubation) (Fernandez-Lopez et al., 2017).

#### Production of Free Fatty Acids (FFAs) From Babassu Oil

The free fatty acids (FFAs) of babassu oil were obtained according to (Mulinari et al., 2017), with some modifications. Shortly, 100 g of oil and an ethanolic solution of KOH (6:1, alcohol/oil) were heated to 80°C, within 1 h under constant mechanical stirring. The reaction took place in a system formed by a condenser coupled to a 500 mL round bottom reaction flask suspended above a water tank. At the end of the reaction, the mixture was transferred to a separatory funnel and washed with 6 M HCl solution to pH 2.0. The upper oily phase was washed with distilled water until neutral pH (Mulinari et al., 2017).

#### Optimization of the Production of Fatty Acid Ethyl Ester

The production of fatty acid ethyl ester was carried out in a 10 mL flask on a rotary shaker with digital temperature control and agitation (Incubator TE-4200) at 200 rpm. The esterification was performed using the FFAs obtained from the hydrolysis of babassu oil and ethyl alcohol as a substrate, with a molar ratio of 1:1–1:5 (FFAs/alcohol). The reaction was initiated by the addition of 1–9% biocatalyst (Fe_3_O_4_@APTES-RML), carried out for a time ranging from 2 to 6 h and temperature ranging from 30 to 50°C. After the specific reaction time for each test, the acidity index was determined for each experiment. Thus, aliquots of 0.3 g were removed from the supernatant volume of the reaction, diluted in 7.5 mL of ethyl alcohol and added 3 drops of phenolphthalein and then titrated with the sodium hydroxide solution (0.1 M) (Aguieiras et al., 2017; Muanruksa and Kaewkannetra, 2020). The acidity index (AI) was determined according to Equation (1) (Cavalcanti et al., 2018).

$$A\,l\,\left(m\,g\,N\,a\,O\,H/g\right)=\frac{M\,M_{N\,a\,O\,H}\cdot M_{N\,a\,O\,H}\cdot f\cdot V_{N\,a\,O\,H}}{m}$$

In which, *MM*_NaOH_ (g/mol) is the molar mass of NaOH; *M*_NaOH_ (mol/L) is the molarity of the NaOH solution; *f* is the correction factor determined by NaOH standardization; *V*_NaOH_ (mL) is the volume of NaOH spent on the titration; and, *m* (g) is the mass of the sample to be analyzed. The conversion of FFAs to esters was calculated considering the acidity of the sample without biocatalyst (*AI*_B_) and the sample containing the biocatalyst (*AI*_S_), Equation (2) (Cavalcanti et al., 2018).

$$ConversionFFA\left(\%\right)=\frac{A\,I_{B}-A\,I_{S}}{A\,I_{B}}\cdot 100$$

#### Taguchi Method

For this study, an advanced experimental design by the Taguchi method with a standard orthogonal matrix L9 (L9 represent the Latin square and the number of experiments, respectively) was used to examine four factors at three levels in order to the optimize the production of fatty acid ethyl ester. Table 1 shows the four independent factors (biocatalyst, molar ratio, temperature and time) and their corresponding levels.

**TABLE 1:** Experimental design coded levels and independent parameters range.

	Temperature (°C)	Time (hours)	Molar ratio (FFAs/alcohol)	Biocatalyst content (%m/m)
Level 1 (L1)	30	2	1:1	1
Level 2 (L2)	40	4	1:3	5
Level 3 (L3)	50	6	1:5	9

Statistica^®^ 10 software was used for experimental design and statistical analysis. Table 3 presents the experimental design together with the conversions and calculated S/N (signal-to-noise) ratios. The values of the S/N ratios corresponding to the conversions values were calculated using the characteristics of the “greater-is-better” function, since the purpose of this study is to maximize the response (conversion). The value of the S/N ratio for each experiment was calculated according to Equation (3).

$$S/N=-10\,\log\left(1/n\,\sum_{i=1}^{n}1/y_{i}^{2}\right)$$

In which, *y*_i_ represents the response variables, *i* being the number of repetitions and *n* representing the number of experiments for the combination of factor levels for any combination of planning. Using Equation (4), it was possible to determine the expected S/N ratio for ideal conditions to obtain the maximum predicted conversion (Chakraborty and RoyChowdhury, 2013).

$$S/N_{p\,r\,e\,d\,i\,c\,t\,e\,d}=\bar{S}/N\,\sum_{j=1}^{n}\left(S/N_{j}-\bar{S}/N\right)$$

In which, $\bar{S}/N$ is the arithmetic mean of all S/N ratios, S/N_j_ is the S/N ratio at the optimal point for each factor and n is the number of factors that significantly affect the process.

#### Gas Chromatography (GC) Analysis

The conversion of ethyl esters was carried out according to the standard EN 14103, with some modifications. Approximately 50 mg of biodiesel was weighed in a 2 ml bottle, and 1 ml of the methyl nonadecanoate solution (10 mg/ml) was added. One microliter of the sample was removed using a syringe (10 μL) and injected into the gas chromatograph (VARIAN-GC 450) with flame ionization detector, column (DB-WAX) – phase: polyethylene glycol, dimensions 60 m long × 0.32 mm internal diameter × 0.25 μm film thickness (Moreira et al., 2020).

## Results and Discussion

### Immobilization Parameters

The immobilization parameters were evaluated after 1 h of immobilization using an enzymatic load of 10 mg of protein per g of support for the hydrolysis of *p*-NPB (50 mM). For all biocatalysts produced, a reference enzyme solution was prepared (an enzyme solution prepared under conditions similar to immobilization, but in the absence of support), the complete activity was maintained during all immobilization tests, allowing the immobilization yield to be calculated by reducing the activity in the supernatant. For the immobilization by adsorption (Fe_3_O_4_@APTES-RML), the immobilization yield was 94.7%, the theoretical activity was 458.0 U/g and the real derivative activity was 341.3 U/g, this allowed an activity of 74.5%, as can be seen in Table 2, which contains the values of the immobilization parameters for the other biocatalysts.

**TABLE 2:** Immobilization parameters of RML: immobilization yield (IY), theoretical biocatalyst activity (At_T_), biocatalyst activity (At_B_), and recovery activity (At_R_) (enzyme loading: 10 mg of protein per 0.1 g of support; 25 mM sodium phosphate at pH 7 and 25°C).

Biocatalyst	IY (%)	At_T_ (U/g)	At_B_ (U/g)	At_R_ (%)
Fe_3_O_4_@APTES-GLU-RML	91.9 ± 0.2	498.5 ± 0.2	199.6 ± 3.5	40.0 ± 3.5
Fe_3_O_4_@APTES-RML	94.7 ± 1.0	458.0 ± 1.0	341.3 ± 1.2	74.5 ± 1.2

*Further details are given in section “Materials and Methods.”*

The immobilization process via adsorption (Fe_3_O_4_@APTES-RML) is favored by the properties of APTES. APTES has a high amino group density (Bini et al., 2012). In this regard, the formation of H-bonded amino groups is favored by APTES due to the smaller number of condensing groups. Thus, the free amino group can react with the surface, and intermolecular hydrogen bonds can be formed (Bruce and Sen, 2005). The zeta potential (ζ) of the amino-functionalized samples can vary considerably with pH. At lower pH values, the ζ-potential increases with the increase in amino group density, which correlates well with the surface concentration of the bonded nanoparticles (Fe_3_O_4_) (Bini et al., 2012). This increase in the positive surface charge provided by the surface amino groups also results in a shift of the isoelectric point to higher pH values (Xu et al., 1997). APTES has an isoelectric point at pH 10.05 (Xu et al., 1997). The interaction between APTES and lipase during the immobilization process occurs at the solid-water interface and is postulated to involve a combination of electrostatic attraction and hydrophobic interaction (Ghiaci et al., 2009; Tzialla et al., 2010; Wang et al., 2019).

The results indicated that RML could efficiently immobilize on APTES surfaces over a broad pH range, with the optimum value being at the physiological condition of pH 7.0. The results suggest that APTES surfaces have sufficient contact sites to bind RML molecules. It is considered that a higher lipase load makes lipase an intermolecular steric obstacle, which restricts the diffusion of the substrate and the product. As a result, relative activity may slowly decrease in contractions greater than 10 mg of protein per g of support. Furthermore, the binding sites on the APTES surface are limited and the enzyme molecules need enough space to catalyze the substrate reaction (Gomes et al., 2004; Wang et al., 2019).

The immobilization performance was also evaluated for the glutaraldehyde-activated supports. However, especially for derivative activity, the non-activated support performed better than one activated with glutaraldehyde. Therefore, Fe_3_O_4_@APTES-RML (RML-MNP, for short) has been used for further characterization and application in this communication.

The activation of the support with glutaraldehyde generates a high concentration of aldehyde groups on the support surface (Adriano et al., 2008; Rodrigues et al., 2008; Mendes et al., 2011; Bonazza et al., 2018). The aldehyde groups in the support and amine groups in the enzyme of the lysine residues are a good option to make the multipoint bond and, therefore, obtain highly thermostable enzymatic derivatives. Glutaraldehyde has low stability in alkaline pH, knowing that immobilization was performed at pH 7. Furthermore, since these lipase preparations have a great tendency to yield bimolecular aggregates. In addition, it has been shown that immobilized lipase activity in aqueous and anhydrous media can be improved in the presence of detergents probably due to the breakage of lipase aggregates and/or to the shift on the closed-open equilibrium of the individual lipase molecules (Mendes et al., 2011). In this way, the application of the immobilization procedure under conditions of dissociation (for example, in the presence of detergents) may allow to obtain fully dispersed immobilized lipase molecules, oriented toward the immobilization system (Palomo et al., 2005). The use of detergents in enzyme immobilization is related to the effect it causes in the process, romping the bonds between enzyme dimers, providing an improvement in the reaction speeds (dos Santos et al., 2015c; Meryam Sardar, 2015). However, according to Fernandez-Lorente et al. (2007), the hydrophobic portion of the detergent can interact with the active lipase center. As a consequence, the detergent can behave as a competitive inhibitor and, therefore, decreasing the values of the derivative’s activities and recovering (Fernandez-Lorente et al., 2007). In the presence of surfactant, immobilization yields were lower, although without much statistical significance.

The Fe_3_O_4_@APTES-GLU-RML fractions were poorly adsorbed, although the immobilization was predominantly by covalent bonding, they may have been desorbed from the support in the presence of the surfactant, thus decreasing the immobilization yield (Palomo et al., 2008; Barbosa et al., 2012). After immobilization, prepared biocatalysts were incubated in sodium borohydride solution to reduce Schiff’s bases (C=N double bond), formed between the aldehyde group of the amine and support and enzyme groups (Rodrigues et al., 2008). The reduction of these Schiff bases turns them into stable covalent bonds, in addition to changing the reactive aldehyde groups to the inert hydroxyl groups, which is an important step in the immobilization process (Rodrigues et al., 2008). Furthermore, the support can also immobilize enzymes, even if they are activated very weakly, because the enzyme is covalently attached to the support at only one point since the glutaraldehyde-protein bonds are stable (Barbosa et al., 2014).

It can be seen in this study that immobilization without the presence of Triton X-100 allowed the formation of the derivative with high catalytic activity and recovered activity, presenting values of 94.7% ± 1.0 and 458.0 ± 1.2 U*p*-NPB/g, respectively. That is, the presence of the detergent acted negatively in the enzymatic immobilization, since it can help in the desorption of the enzyme from the support, in addition, it can act as an inhibitor (it acts as if it were the enzyme-substrate).

Immobilization of *Rhimozucor miehei* lipase in different supports has been reported in several studies in the literature. Mohammadi et al. (2014), stated in their work with immobilization of RML by adsorption method on silica nanoparticles (MCM-41), porous support, and functionalized with glycidyloxypropyltrimethoxysilane, that after 24 h of incubation in 25 mM sodium phosphate buffer solution at pH 7.0 and 25°C, they achieved an immobilization yield of 52%. The immobilization occurred mainly on the exterior of the particles, probably the small pore size (3.9 nm) of the support is not the most adequate for the internal surface to become more accessible for the lipase immobilization. Other authors analyzed the immobilization of RML in functionalized aldehyde-agarose (Gx-RML), also by adsorption, and obtained an immobilization yield of 47%, after 20 h of incubation at pH 10 at 4°C. This low immobilization yield is linked to severe immobilization conditions (time and pH). As a result, the amount of Lys groups on the enzyme surface was reduced, which limited the multipoint binding of the enzyme and the support (Yousefi et al., 2020). Thus, the MNPs magnetic nanoparticles used in this communication, have a special place as support matrices and versatile carriers for immobilization protocols, due to their large surface area, high mass transfer, and large surface/volume proportions. One of the main bottlenecks for the biotechnology sectors is the lack of efficient purification and recovery of enzymes from the reaction media. Biocatalysts immobilized into MNPs could be easily recovered by the application of an external magnetic field, extending the biocatalyst life through several recovery cycles (Bilal et al., 2018; Zhong et al., 2020).

### Characterization of the Biocatalysts

**FIGURE 1:**
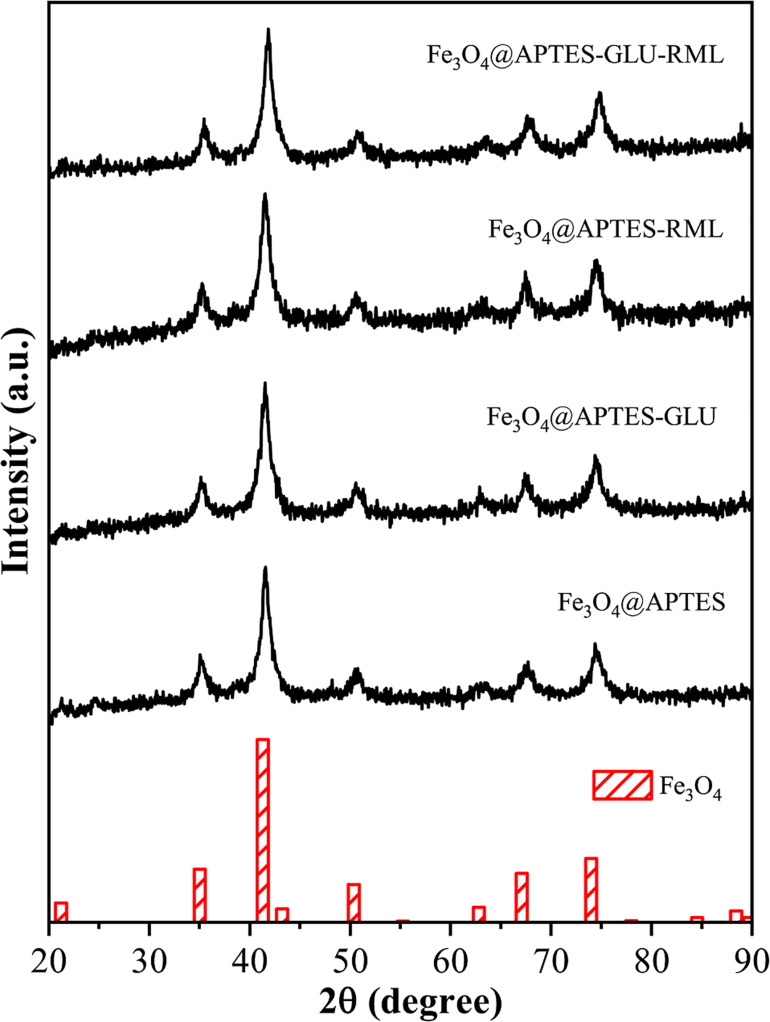
XRPD patterns of the Fe_3_O_4_@APTES before and after chemical and physical immobilization of RML and reference pattern of Fe_3_O_4_, JCPDS 01-086-1358. Other specifications are described in section “Materials and Methods.”

Taking into consideration the immobilization results obtained through the Fe_3_O_4_@APTES support, additional characterizations of the samples were carried out in order to investigate their structural, magnetic and chemical properties. The structure and phase composition of the support were confirmed by XRPD, as shown in Figure 1. The samples showed broad peaks at *2*θ of 21.3, 35.2, 41.5, 50.7, 63.0, 67.5, 74.3 84.6, and 89.1°, which can be attributed (111), (220), (311), (400), (422), (511), (440), (620) and (533) planes, respectively, of a spinel structure, related to Fe_3_O_4_ (JCPDS 01-086-1358). No other crystalline phase was observed. Furthermore, the XRD patterns before and after chemical and physical immobilizations are similar, indicating the presence of glutaraldehyde and lipase in the nanocomposites does not affect the structure of the support.

**FIGURE 2:**
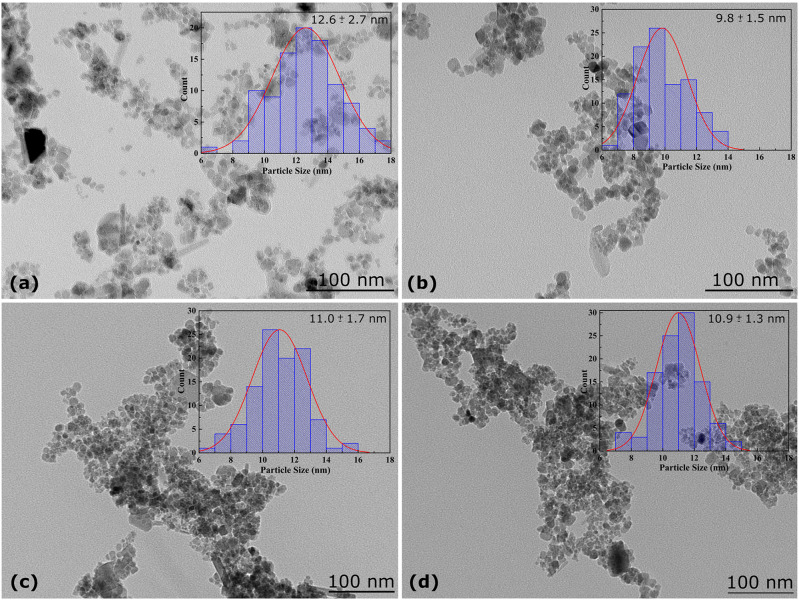
TEM images of the samples studied: **(a)** Fe_3_O_4_@APTES, **(b)** Fe_3_O_4_@APTES-GLU, **(c)** Fe_3_O_4_@APTES-GLU-RML, and **(d)** Fe_3_O_4_@APTES-RML. The inset in each micrograph displays the distribution size graph built based on the measurement of 100 NPs randomly chosen. Other specifications are described in section “Materials and Methods.”

In order to investigate the size, as well as the morphology of the biocatalyst, TEM was carried out. The micrographs for each sample are displayed in the Figure 2. The inset in each micrograph presents the distribution size graph related to the sample. To build this graph, 100 NPs from different regions of the TEM grid were randomly chosen and measured. To obtain the size of these truncated nanomaterials, the feret’s statistical diameter was used to measure each NP. Also, a log-normal function to fit the data and the average diameter were found to be 12.6 ± 2.7, 9.8 ± 1.5, 11.0 ± 1.7, and 10.9 ± 1.3 nm for Fe_3_O_4_@APTES, (b) Fe_3_O_4_@APTES-GLU, (c) Fe_3_O_4_@APTES-GLU-RML and (d) Fe_3_O_4_@APTES-RML, respectively. Taking these values into consideration, the average diameters of the nanomaterials can be assumed to be statistically equals. Furthermore, no regular morphology was observed.

**FIGURE 3:**
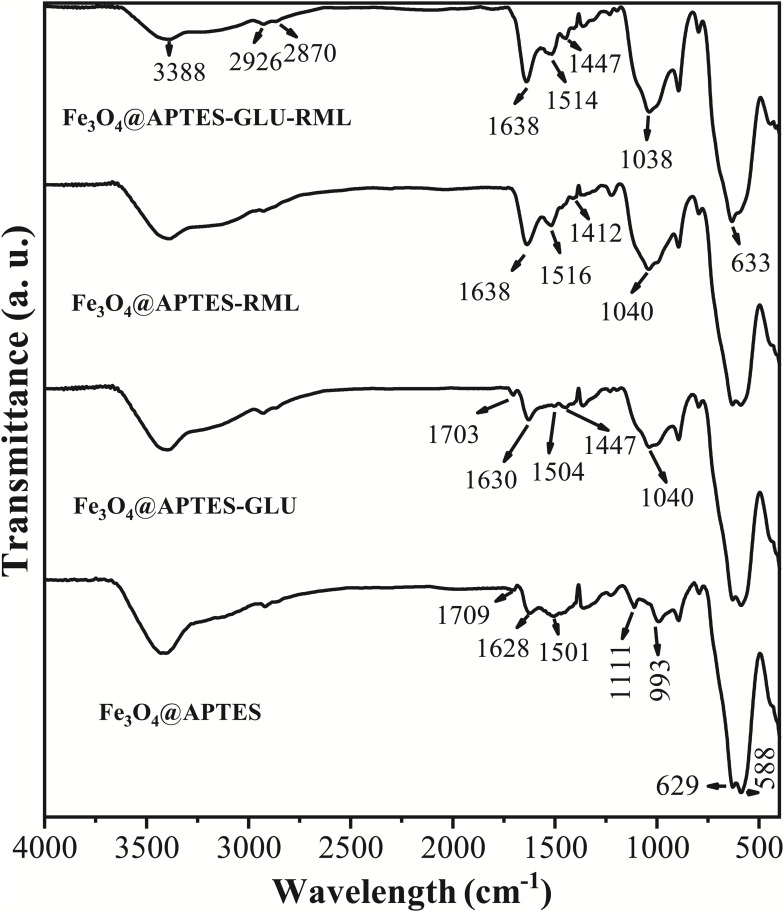
FTIR spectra of Fe_3_O_4_@APTES NPs before and after chemical and physical immobilization of RML. Other specifications are described in section “Materials and Methods.”

Figure 3 shows the FTIR spectra of the Fe_3_O_4_@APTES support before and after the chemical bonding, as well as physical adsorption of RML. All these samples showed bands around 3388 and 1638 cm^–1^, assigned to overlapped stretching vibrations of hydroxyls and N-H groups and overlapped bending vibrations of adsorbed H_2_O and free amino groups on the surface of the nanoparticles, respectively (Karimi et al., 2016; Shafiee et al., 2019). The bands observed around 629 and 588 cm^–1^ are characteristic of Fe-O vibrations in γ-Fe_2_O_3_ and Fe_3_O_4_ phases, indicating that the synthesized materials consist of partially oxidized Fe_3_O_4_, as evidenced in XRPD (Neto et al., 2017). The support functionalization was evidenced by the presence of bands at 2926, 2870, 1111, and 993 cm^–1^, which can be attributed to asymmetric and symmetric C–H stretching of –CH_2_ and Si-O–H and Si-O stretching vibrations, respectively (Shafiee et al., 2019). After reaction with glutaraldehyde, the bands at 1709 (C=O stretching of aldehyde group) and 1503 (N-H bending) cm^–1^ were found to change their shape, as expected with the modification of amino groups from APTES to leave free aldehyde groups (Karimi et al., 2016). The efficacy of RML immobilization was evidenced by the increase in the relative intensity of the bands at 1657, 1535, and 1458 cm^–1^, assigned to C=O stretching [amide (I), N–H bending (amide (II)] and symmetric bending from saturated C–H, respectively (Abdul Manan et al., 2018; Zhang et al., 2020). The chemical immobilization in the Fe_3_O_4_@APTES-GLU-RML sample was confirmed by the disappearance of aldehyde band (1709 cm^–1^) in the spectra. Furthermore, shifts of characteristic peaks of Fe_3_O_4_@APTES and Fe_3_O_4_@APTES-GLU suggests the existence of electrostatic interactions and hydrogen bonding between these supports and RML.

**FIGURE 4:**
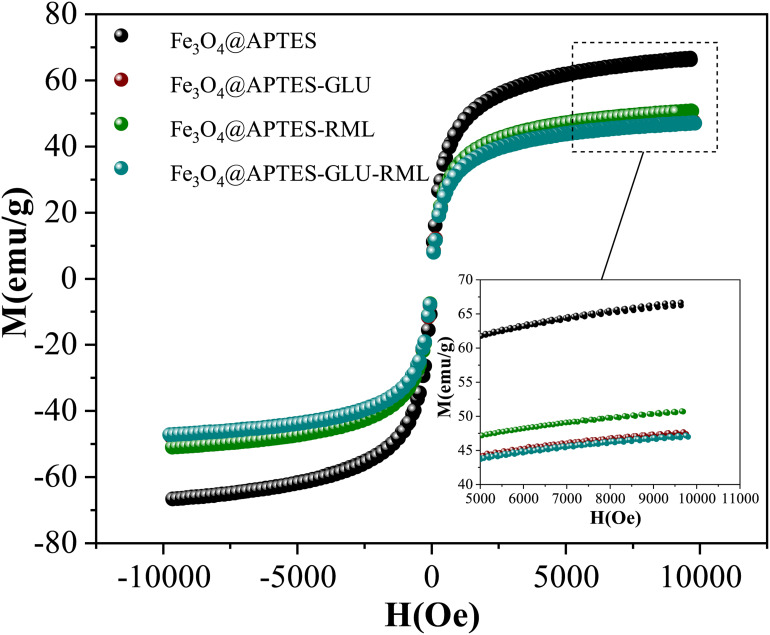
Magnetization curves at 300 K of Fe_3_O_4_@APTES NPs before and after chemical and physical immobilization of RML. Other specifications are described in section “Materials and Methods.”

Figure 4 shows the magnetization curves at room temperature for the materials obtained with Fe_3_O_4_@APTES support. No hysteresis was observed for the samples, which evidencing the superparamagnetic nature. The values of saturation magnetization (*M*_s_) were found to be 66.70, 50.76, 47.69, and 47.00 emu/g for Fe_3_O_4_@APTES, Fe_3_O_4_@APTES-RML, Fe_3_O_4_@APTES-GLU, and Fe_3_O_4_@APTES-GLU-RML, respectively. These values are smaller than the one reported for bulk Fe_3_O_4_ (92 emu/g). However, this may be explained based on the presence of surface spin disorders (dead layer) as the particle size decreases (Wang et al., 2016; Sharifi Dehsari et al., 2018). Moreover, another significant contribution comes from the existence of non-magnetic materials attached to the surface of the magnetic support. In this regard, it is worth to note the smaller *M*_s_ values for materials obtained after chemical modification and immobilization supports that the functionalization and immobilizations were successful.

### Effect of pH

**FIGURE 5:**
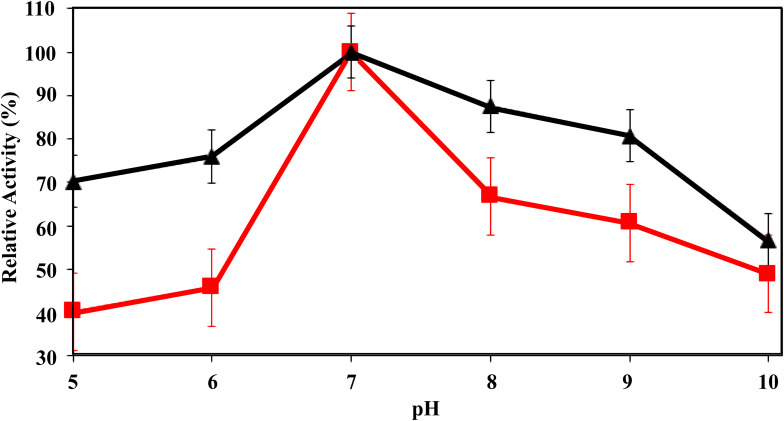
Effect of the pH value on *p*-NPB activity of RML (red squares) and Fe_3_O_4_@APTES-RML (black triangles). Further details are given in section “Materials and Methods.” One hundred percentage is considered the activity of the free enzyme at pH 7 (optimal conditions for the enzyme) and correspond to around 450 U/mg. Other specifications are described in section “Methods.”

All biocatalysts, free and immobilized lipase were resuspended at different pH values of 25 mM buffer in the pH range ranging from 5 to 10 [sodium acetate (pH range 3.6–5.6), sodium phosphate (pH range 5.8–8.0) and sodium carbonate (pH range 8.9–10.8)]. A very important parameter in the preparation of active biocatalysts is the immobilization pH, as it influences the degree of ionization of the protein molecules and the surface charge of the supports (Huang et al., 2006). The effect of pH on the performance of the soluble and immobilized biocatalysts was evaluated by analyzing the activity in the range of pH 5–10, a limit range for substrate stability. In Figure 5, it is possible to observe that soluble and immobilized RML showed maximum activity at pH 7. Other authors analyzed the effect of pH on RML activity and obtained the same profile presented in this study, in which the greatest activity was at pH 7 (Adamczak and Bednarski, 2004; de Oliveira et al., 2018).

Both, soluble and immobilized RML, presented a similar behavior at acidic and basic pHs values. As a matter of fact, it was possible to observe a lower activity at more acidic pHs values (pHs 5 and 6) and a higher activity at more alkaline pHs values (pHs 8 and 9). However, the activity of soluble and immobilized RML decreases as the pH value approaches the pH 10. This reduction may be linked to the fact that this is a pH range that is not suitable for RML, thus compromising the stability of the biocatalyst (Rodrigues and Fernandez-Lafuente, 2010; de Oliveira et al., 2018).

Based on the literature, immobilization pH alters the balance between positive and negative charges on the enzyme surface (de Albuquerque et al., 2016; Machado et al., 2019), thus generating possible electrostatic repulsion between lipase molecules and the ion-exchange support at alkaline pH values. The results show that the pH of the immobilization can change the orientation of the enzyme in the prepared support, that is, its characteristics, for example, the catalytic activity (Barbosa et al., 2014; Machado et al., 2019). Immobilization at pH 7 is important due to a greater reactivity of amino terminal groups than the reactivities of all amino groups Lys at neutral pH values (Mateo et al., 2005). However, at pH 6–7, covalent bonds of enzymatic support may occur between the different groups available in high concentration on the support and some nucleophiles of proteins that are in the area exposed to the support (Bolivar et al., 2009). In addition, pH can enhance the denaturation of enzymes that have resulted in decreased activity (Garcia-Galan et al., 2011). On the other hand, the enzyme’s catalytic activity depends on the protein’s conformational structure, even small changes in the protein’s tertiary structure resulted in the loss of its catalytic activity (Ansari and Husain, 2011).

A factor that influences the immobilization process is the isoelectric point of the enzyme. At this point, the maximum hydrophobic interaction between the lipase and the support surface can occur, which is called the interfacial activation mechanism, which benefits the enzymatic adsorption process and the positioning of the substrate molecules outside the biocatalyst (Teodoro et al., 2019). The isoelectric point of the lipase from *Rhizomucor miehei* is approximately 3.8 (Rodrigues and Fernandez-Lafuente, 2010). This effect of the isoelectric can be seen in Figure 5. From pH 5, it is possible to observe the interaction between lipase and support, for immobilized lipase in relation to lipase in its soluble form, proving the mechanism of interfacial activation of the immobilization process (Teodoro et al., 2019).

### Thermal Stability

Soluble and immobilized RML were analyzed for thermal and pH inactivation, at a temperature of 60°C. The stability of Fe_3_O_4_@APTES-RML was higher at pH 7 (t_1/2_ = 108 min), followed by pH 5 (t_1/2_ = 105 min) and the lowest was at pH 10 (t_1/2_ = 91 min), while RML showed greater stability at pH 7 (t_1/2_ = 12.8 min), followed by pH 5 (t_1/2_ = 6.5 min) and pH 10 (t_1/2_ = 5.8 min) (see Table 3). The rapid thermal deactivation of soluble RML has been reported in the literature (de Oliveira et al., 2018; Rahman et al., 2018). Therefore, the immobilization of RML on Fe_3_O_4_@APTES was able to increase its thermostability. This may be related to the surface properties of the support and the immobilization method (Cui et al., 2013). In addition, the microenvironment between enzyme and support may be another important reason (Cui et al., 2013). When the enzyme is immobilized, the affinity of the support for water can influence its catalytic activity.

**TABLE 3:** Half-life for RML and Fe_3_O_4_@APTES-RML at 60°C and pHs 5, 7, and 10.

Biocatalyst	Half-life (t_1/2_, min)		
	pH 5	pH 7	pH 10
RML	6.4	12.8	5.8
Fe_3_O_4_@APTES-RML	105	108	91

*Further details are given in section “Materials and Methods.”*

In this work, another possible reason for the enhanced thermal stability of the lipase immobilized on the amino-modified solid support may be due to a change in the microenvironment around the enzyme due to the presence of amino groups (Bolivar and Nidetzky, 2019). As a cationic polymer, APTES has been used to adsorb enzymes and stabilize proteins in solution, preventing oxidation, aggregation, and supports coated with APTES have been used to stabilize multimeric enzymes, preventing the dissociation of subunits (Fernandez-Lafuente, 2009; Aissaoui et al., 2013). As a result, a cationic polymer and amino group provider could immobilize lipase by ion exchange. Then, the lipase could be immobilized on support by covalent bonding and ion exchange by multipoint immobilization, which was more stable than immobilization by covalent bonding alone (Godoy et al., 2011). Furthermore, the secondary structure of the lipase was more integrated into the cationic polymer. Therefore, surface modification with a cationic polymer would be beneficial to improving the activity and stability of the immobilized enzyme as well as increasing the loading amount (Cowan and Fernandez-Lafuente, 2011; Tian et al., 2016).

Furthermore, temperature may cause the unfolding of the RML tertiary structure, which may result in the modification of its active site and cause the deactivation of lipase (Dave and Madamwar, 2006). However, the immobilization of RML may increase the rigidity of the biocatalyst, becoming less susceptible to conformational changes caused by increases in temperature; besides, the immobilization of RML may have stabilized the lipase in its open conformation, which cause an increase in enzymatic activity (de Oliveira et al., 2018; Rahman et al., 2018).

### Optimization of the Production of Fatty Acid Ethyl Ester

As can be seen in Table 4, run 6 showed the highest conversion to ethyl ester and S/N ratio, using 5% of biocatalyst content and 1:1 molar ratio (FFAs/alcohol), in 6 h of reaction under 40°C. Using the “greater is better” function, it was possible to determine the levels of the reaction variables for optimized fatty acid ethyl ester production. As can be seen in Table 5, the optimal reaction levels were L1 (30°C) for the reaction temperature, L2 (4 h) for reaction time, L1 (1:1) for the molar ratio (FFAs/alcohol) and L3 (9%) for the content of biocatalyst; under these conditions, the theoretical conversion is 93.4%. These optimal conditions were validated by chromatographic analysis of ethyl esters, following the standard EN 14103 with some modifications, the value obtained was 78.9% ± 0.0%. The optimized result found through the chromatographic analysis was less than the theoretical value of the conversion proposed by the method. It is worth mentioning that because it is a biological process, the result is susceptible to interference from several factors. Short-chain alcohol and vegetable oils form a solution in which the molar ratio is approximately 1:1 (at a temperature of 40°C). When alcohol is insoluble in the reaction, emulsion formation occurs and the size of the particles depends on the intensity of the agitation. As a result, the biocatalyst may undergo inactivation. By an adding organic solvent to the solution, the solubility of the alcohol increases, protecting the enzymes from inactivation and ensuring that conversion to esters can occur (Szczêsna Antczak et al., 2009). The authors (Shieh et al., 2003), evaluated the production of biodiesel from soybean oil, using the Lipozym RM commercialized immobilized version of the lipase from *Rhizomucor miehei* in the transesterification reaction. The authors obtained a conversion of 92.2% in 6.3 h time, with a molar ratio of 3.4:1 (methanol:oil) at a temperature of 37°C (Shieh et al., 2003). However, the value of 78.9% ± 0.0% conversion to fatty acids of ethyl esters obtained in the present communication using lipase from *Rhizomucor miehei* immobilized onto magnetic nanoparticles by adsorption, was reached in 4 h with a temperature of 30°C, milder reaction conditions and less energy consumption.

**TABLE 4:** Taguchi method for the oil from babassu esterification reaction, orthogonal matrix (L9).

Run	Temperature (°C)	Time (h)	Molar ratio (FFAs/alcohol)	Biocatalyst (%m/m)	Conversion (%)	S/N
1	30	2	1:1	1	14.6 ± 0.5	23.3
2	30	4	1:3	5	45.1 ± 0.1	33.1
3	30	6	1:5	9	45.3 ± 0.0	33.1
4	40	2	1:3	9	50.5 ± 0.1	34.1
5	40	4	1:5	1	6.9 ± 0.6	16.8
6	40	6	1:1	5	81.7 ± 0.7	38.2
7	50	2	1:5	5	10.2 ± 0.6	20.2
8	50	4	1:1	9	79.8 ± 0.7	38.0
9	50	6	1:3	1	5.4 ± 0.2	14.7

*Additional details are provided in section “Materials and Methods.”*

**TABLE 5:** S/N ratios response.

Levels	Temperature (°C)	Time (h)	Molar ratio (FFAs/alcohol)	Biocatalyst (%m/m)
1	29.8	25.8	33.2	18.3
2	29.7	29.3	27.3	30.5
3	24.3	28.7	23.3	35.1
Delta	5.5	3.5	9.8	16.8
Ranking	3	4	2	1

*Further details are given on the section “Materials and Methods.”*

According to Resolution No. 51 of the Brazilian National Agency of Petroleum, Natural Gas and Biofuels (ANP), conversion into esters needs to present a minimum value of 96.5% to be considered as biodiesel. As the maximum value obtained in the present study was 81.7% ± 0.7, in experiment 6 of Table 2, it was not possible to produce biodiesel in the present study.

In studies developed by Mohammadi et al. (2015), the authors immobilized lipase from *Rhizomucor miehei* on two supports: silica (silica-RML) and silica nanoparticles (SBA-RML). These biocatalysts were applied in the transesterification reaction of rapeseed oil with methanol for the synthesis of methyl esters in a solvent-free system. For silica-RML, the conversion yield was 43% after 72 h of incubation at 50°C for 200 mg of silica-RML and a 3:1 molar ratio (methanol:oil). The conversion yield for the SBA-RML was 28%, under the same reaction conditions previously reported (Mohammadi et al., 2015). Other authors have evaluated the synthesis of ethyl esters from soybean oil by lipase from *Rhizomucor miehei* immobilized in ZIF-8 by the encapsulation method; as a result, it showed a conversion of 84.7% after 17 h of incubation at 45°C to 6% wt of biocatalyst (RML@ZIF-8) and a molar ratio of 1:4 (oil:alcohol) (Adnan et al., 2018). Therefore, the optimized results obtained in this communication for the synthesis of fatty acids of ethyl esters of babassu oil showed higher values when compared to some biocatalysts presented in the literature under milder reaction conditions. However, compared to the biocatalyst that was immobilized by the encapsulation methodology, the RML-MNPA exhibited a slightly lower result, but still under conditions of milder reactions. This would be linked to the fact that in the immobilization methodology by adsorption is a weaker procedure than the encapsulation because easier desorption of the support enzyme may occur (Adnan et al., 2018; Boudrant et al., 2020).

**FIGURE 6:**
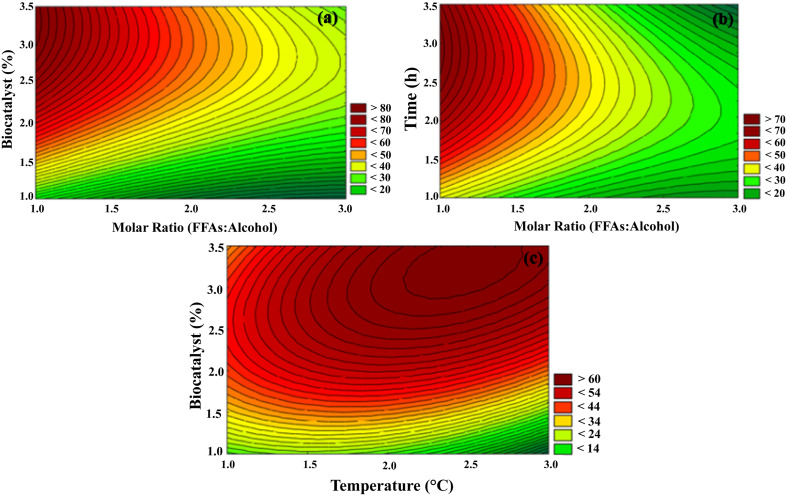
Contour surfaces for biodiesel production. **(a)** Ratio Molar (FFAs/alcohol) vs. Biocatalyst (%). **(b)** Ratio Molar (FFAs/alcohol) vs. Time (h). **(c)** Temperature (°C) vs. Biocatalyst (%). Other specifications are described in section “Methods.”

Based on Table 5 and Figure 6a, the most significant variable for the production of fatty acid ethyl ester from free fatty acids of babassu oil was the content of the biocatalyst. In fact, the amount of biocatalyst is an important parameter to determine the fatty acid ethyl ester yield; as the concentration of biocatalyst is high, the yield of fatty acid ethyl esters increases, as more substrate molecules will adsorb to the active site of the lipase (Jegannathan et al., 2010; Duraiarasan et al., 2016).

In Table 3 and Figure 6b, as can be seen, the molar ratio at its lowest level positively influenced fatty acid ethyl ester production. It is important to note that the conversion value is influenced by the properties of the substrate and the nature of the catalyst (Madras et al., 2004). Alcohols may perform two roles in esterification reactions. The first is related to the excess of alcohols that can increase the reaction rate and boost high yield. Second, a high concentration of alcohol might negatively affect enzymes, which generally make them more unstable in alcohol, such as methanol and ethanol. Thus, the deactivation of the biocatalyst through contact with soluble alcohol present in a reaction results in reductions in the production of ethyl or methyl ester (Adnan et al., 2018). It can be seen that the best conversion to ethyl esters was found in the 1:1 molar ratio and that it did not significantly increase the conversion value in larger molar ratios. Lipozyme RM-IM was studied, for the production of fatty acid ethyl ester using fish oil and ethanol as a substrate, the authors concluded that the highest yields were found in the lowest 1:0.25 molar ratio (fish oil/ethanol), showing the low resistance of the enzyme to the presence of alcohol (Marín-Suárez et al., 2019). On the other hand, unlike the other parameters, time did not influence the conversion values in the interval between 2 and 6 h, as shown in Table 5. However, based on Table 4, the greatest conversion was found in the longest time (6 h). Conversions to fatty acid ethyl esters can be increased in longer reaction times (Maceiras et al., 2009).

Based on Figure 6c and Table 5, it is possible to observe that the milder temperatures played a positive role in the production of fatty acid ethyl ester. Higher temperatures lead to an extra cost for any industrial process and may cause the denaturation of enzymes, causing a reduction in enzyme efficiency (Fjerbaek et al., 2009). In fact, for reaction catalyzed by lipases, the reaction rate may be improved with increasing temperature until a certain level, once at high temperatures, lipases may undergo denaturation (Wu et al., 2003).

The data of the conversion of free fatty acids into ethyl esters optimized by the Taguchi method were statistically analyzed using the Analysis of Variance (ANOVA) presented in Table 6. To identify the most significant process parameter for the conversion, it was necessary to determine the percentage contribution of each factor. This percentage contribution of the parameters was calculated based on the average of the % of conversion and the estimated S/N ratio. Among the parameters studied, only the biocatalyst content had a significant effect on conversion (*p* < 0.05), the other parameters did not show a statistically significant result. As a result, the biocatalyst content was the factor that exerted the greatest influence on conversion (contribution of 66.6%), confirming the results shown in Table 5 and Figure 6.

**TABLE 6:** ANOVA for parameters that affect the esterification of babassu oil. Insignificant factorial effect is grouped as shown {}.

Factor	DF	SS	MS	*F*-value	*p*-value	Contribution (%)
Temperature	2	59.5	29.7	2.9	0.257^*a*^	8.7
Time	{2}	{20.5}	–	–	–	3.0
Ratio molar	2	147.2	73.6	7.2	0.123^*a*^	21.6
Biocatalyst	2	453.9	226.9	22.1	0.043^*a*^	66.6
Residual	2	10.2				
Total	8	670.8				100

*Further details are given on the section “Materials and Methods.” ^a^Means a 95% confidence interval reflects a significance level < 0.05.*

## Conclusion

The immobilization of lipase from *Rhizomucor miehei* (RML) onto magnetic nanoparticles coated with 3-aminopropyltriethoxysilane produced the biocatalyst Fe_3_O_4_@APTES-RML (RML-MNPA). The biocatalyst was analyzed for the immobilization parameters obtained (94.7% ± 1.0 for immobilization yield and 341.3 ± 1.2 U/g for derived activity), thermal and pH deactivation that resulted in a time of 16 times longer half-life for immobilized enzyme (RML-MNPA) compared to free RML. The performance of the immobilization protocol used was confirmed by FTIR, XRPD and SEM analyzes. The RML-MNPA was studied and optimized in the synthesis of fatty acid ethyl ester from fatty acids from babassu oil, under optimized reaction conditions to increase the conversion into fatty acid ethyl esters. The reaction conditions were determined using the Taguchi methodology, in which it was possible to obtain a conversion of 81.7 ± 0.7% in the conditions (5% w/w) of RML-MNPA, 1:1 (FFAs/alcohol), 40°C and 6 h). Thus, RML-MNPA is an alternative in the production of fatty acid ethyl esters, as it has specificity for the substrate and can be easily recovered from the reaction when exposed to a magnetic field.

## Data Availability Statement

The raw data supporting the conclusions of this article will be made available by the authors, without undue reservation, to any qualified researcher.

## Author Contributions

JS and KM: conceptualization and investigation. JS, KM, MS, LJ, RM, and AO: methodology. KM and LJ: software. KM: validation and writing – original draft preparation. KM, PF, FM, LF, JD, SM, RF, and PF: formal analysis. JS, PF, and MS: resources and funding acquisition. PF: data curation. AO and PF: writing – review and editing. JS, RM, and AO: visualization. JS and MS: supervision. JS and PF: project administration. All authors contributed to the article and approved the submitted version.

## Conflict of Interest

The authors declare that the research was conducted in the absence of any commercial or financial relationships that could be construed as a potential conflict of interest.
